# Development of a Parenteral Formulation of NTS-Polyplex Nanoparticles for Clinical Purpose

**DOI:** 10.3390/pharmaceutics10010005

**Published:** 2018-01-03

**Authors:** María E. Aranda-Barradas, Maripaz Márquez, Liliana Quintanar, Jaime Santoyo-Salazar, Armando J. Espadas-Álvarez, Daniel Martínez-Fong, Elizabeth García-García

**Affiliations:** 1 Nanosciences and Nanotechnology Department, Center for Research and Advanced Studies of the National Polytechnical Institute, Mexico City 07360, Mexico; mariaaranda500@gmail.com (M.E.A.-B.); martinez.fong@gmail.com (D.M.-F.); 2 Chemistry Department, Center for Research and Advanced Studies of the National Polytechnical Institute, Mexico City 07360, Mexico; maripaz.marquez@gmail.com (M.M.); lilianaq@cinvestav.mx (L.Q.); 3 Pharmacology Department, Center for Research and Advanced Studies of the National Polytechnical Institute, Mexico City 07360, Mexico; 4 Physics Department, Center for Research and Advanced Studies of the National Polytechnical Institute, Mexico City 07360, Mexico; sjimmyster@gmail.com; 5 Physiology, Biophysics and Neurosciences Department, Center for Research and Advanced Studies of the National Polytechnical Institute, Mexico City 07360, Mexico; armandoespadas1@gmail.com; 6 Pharmaceutical Nanotechnology Department, Psicofarma, S.A. de C.V., Mexico City 14050, Mexico

**Keywords:** nanoparticles, gene therapy, formulation, lyophilization, transfection, stability

## Abstract

Neurotensin (NTS)-polyplex is a nanoparticle system for targeted gene delivery that holds great promise for treatment of Parkinson’s disease and various types of cancer. However, the high instability in aqueous suspension of NTS-polyplex nanoparticles is a major limitation for their widespread clinical use. To overcome this obstacle, we developed a clinical formulation and a lyophilization process for NTS-polyplex nanoparticles. The reconstituted samples were compared with fresh preparations by using transmission electron microscopy, dynamic light scattering, electrophoretic mobility, circular dichroism and transfection assays in vitro and in vivo. Our formulation was able to confer lyoprotection and stability to these nanoparticles. In addition, transmission electron microscopy (TEM) and size exclusion-high performance liquid chromatography (SEC-HPLC) using a radioactive tag revealed that the interaction of reconstituted nanoparticles with fetal bovine or human serum did not alter their biophysical features. Furthermore, the formulation and the lyophilization procedure guaranteed functional NTS-polyplex nanoparticles for at least six months of storage at 25 °C and 60% relative humidity. Our results offer a pharmaceutical guide for formulation and long-term storage of NTS-polyplex nanoparticles that could be applied to other polyplexes.

## 1. Introduction

Neurotensin polyplex (NTS-polyplex) consists of nanoparticles (NPs) that were functionalized to deliver therapeutic genes into cells that express NTS receptor type 1 (NTSR1) [1]. Those NPs result from a plasmid DNA (pDNA) compaction by electrostatic binding of poly-l-lysine and the VP1 SV40 karyophilic peptide (KP). The KP, the natural ligand NTS and the hemagglutinin-derived HA2 fusogenic peptide (FP) are the molecules that functionalize NTS-polyplex NPs. NTS and FP are covalently linked to poly-l-lysine to form a conjugate known as NTS-carrier. Every molecule that decorates NTS-polyplex NPs stimulates a specific cell function and they act sequentially, to transfer the pDNA, containing the gene of interest (transgene), to the target cells. Thus, NTS in NTS-polyplex NPs enables selective transgene transfer via internalization of NTSR1 that is expressed in the plasma membrane of target cells [2,3]. FP rescues NTS-polyplex from endosomal degradation and KP transports the pDNA to the cell nucleus where the transgene is transcribed [4,5].

The results obtained after an intracranial administration of NTS-polyplex NPs have demonstrated efficient and long-lasting expression of neurotrophic factor genes in dopamine neurons of hemiparkinsonian rats [6,7,8]. Similarly, the intravenous administration leads to efficient transfection of suicide genes into tumor cells in animal models of triple negative breast cancer and neuroblastoma [9,10]. Hence, NTS-polyplex NPs can be a promising gene delivery system for the treatment of these pathologies. However, the instability in physiological solutions is a major limitation of NTS-polyplex NPs to bridge experimental phases in animals to human clinical trials. So, a long shelf life must be achieved to advance NTS-polyplex NPs to clinical use.

Previous studies have characterized some biophysical properties of NTS-polyplex NPs formulated in Dulbecco’s Modified Eagle’s (DMEM) that determine their transfection efficiency. In this characterization, transmission electron microscopy (TEM) and scanning electron microscopy (SEM) analysis have shown that NTS-polyplex NPs have toroid shape, with a diameter of 100–150 nm [3,5,9,11,12,13] at the optimum pDNA:KP:NTS-carrier molar ratio, as determined by their biological functionality in vitro [11]. The toroid shape of polyplexes results from the neutralization of pDNA polyanionic charges by electrostatic interaction with a cationic polymer [14]. This neutralization frequently leads to a strong propensity to aggregate and thus, to a poor stability in aqueous solutions, resulting in decreased transfection efficiency and therapeutic efficacy over time [15,16,17,18].

There are some aspects to be considered in the development of a formulation for clinical purposes that could contribute to overcome these drawbacks. For example, saline concentration influences directly the size of complexes [15,19] and when glucose is included in vehicles, NPs tend to distribute in a homogeneous population, avoiding aggregation, as compared to those prepared in physiological salt solutions [15,20]. Likewise, vehicles containing sodium phosphate provide better transgene expression in vivo than those containing NaCl, or phosphate-buffered saline (PBS) [21].

Moreover, lyophilization is a critical step in the stability and shelf life of polyplexes [15,16,21,22]. Since the functionalization of NTS-polyplex NPs depends on four peptides and pDNA, lyophilization must preserve the structural integrity of all these components to reproduce specificity and transfection efficiency of this nonviral system. Both, the use of a lyoprotectant in the formulation and special conditions in the lyophilization process are required to protect the NPs from damage by freezing and drying stress. Ice formation during freezing can affect particle stability of polyplexes when exposed to an ice-liquid interface, thus causing mechanical damage by ice crystal growing. In addition, cryoconcentration of salts and NPs during drying promotes aggregation because of ionic strength increase, pH shifts and NPs crowding [23]. Saccharides have been used as excipients to protect and prevent aggregation of NPs during the freezing step of lyophilization process. These sugars have the ability to form a protective sugar-glass during freezing of the solution [22] and isolate individual particles in the unfrozen fraction, thereby preventing aggregation during freezing [23]. Also, by effectively substituting for water, carbohydrates can form hydrogen bonds to the surface of macromolecules, allowing NPs to conserve their native structure integrity [24]. Sugar/DNA ratios and, thus, final saccharide concentrations to provide cryo- and lyoprotection depend on the particle concentration [25], the chosen saccharide and on the type of cationic polymer of polyplexes [23]. However, high concentrations of saccharides result in formulations that are not osmotically compatible with cell viability and therefore are not adequate for clinical use [26].

This work aimed to advance the characterization of NTS-polyplex NPs in the experimental formulation (DMEM), by determining the morphology, folding, particle size, zeta potential and tertiary structure that provide high transfection efficiency and functionality in vitro and in vivo. The values established in such characterization were the reference parameters to optimize the formulation prototype for the nonviral system. We also determined the integrity of the clinically formulated and lyophilized NTS-polyplex NPs after their reconstitution and interaction with both fetal bovine serum (FBS) and human serum. Finally, we evaluated the lyophilizates at different storage times and after submitting them to accelerated and long-term stability tests. Our results offer a clinical formulation for long-term storage of functional NTS-polyplex NPs.

## 2. Materials and Methods

### 2.1. Reagents

FP (GLFEAIAEFIEGGWEGLIEGSAKKK-COOH) and KP (Ac-MAPTKRKGSCPGAAPNKPK-COOH) were obtained from RS Synthesis (Lousville, KY, USA). NTS and poly-l-Lysine hydrochloride (30–70 kDa) were obtained from Sigma-Aldrich (St. Louis, MO, USA). Long chain (succinimidyl 6-(3(2-pyridyldithio)propionamido)hexanoate) (LC-SPDP) was purchased from Thermo Scientific (Rockford, IL, USA). Plasmid pEGFP-N1 coding for the green fluorescent protein (GFP) (Clontech; Mountain View, CA, USA) was amplified and purified using MaxiPrep Kit, Qiagen (Manchester, UK) in accordance with the supplier’s instructions.

### 2.2. Synthesis of NTS-Carrier and Formation of NTS-Polyplex NPs

NTS-carrier was synthesized using LC-SPDP as crosslinker following the procedure reported previously [11]. NTS-polyplex NPs were obtained by adding dropwise KP solution to a pDNA solution to form the KP-pDNA complex. The KP optimum final concentration was 5 μM for 6 nM of pDNA according to the criterion of electrophoretic retention assays [2,3,11]. This mixture was homogenized by using a vortex Genie 2 (Scientific Industries; New York, NY, USA), at 900 rpm for 30 min. Then, NTS-carrier solution was slowly added to KP-pDNA complex solution and then vortexed at 900 rpm for 30 min to form the NTS-polyplex NPs. The optimum molar relation of pDNA:NTS-carrier was 1:24 (NTS-carrier 144 nM), according to the criterion of electrophoretic retardation assays [2,3]. As a positive control, NTS-polyplex NPs were assembled in DMEM (GIBCO, Invitrogen; New York, NY, USA), which we have named as the experimental formulation (EF), supplemented with 25 mM glucose (EF25) or 280 nM glucose (EF280). The prototype formulations (PF) for NTS-polyplex NPs contained 136 mM NaCl, 3.7 mM KCl, 1.2 mM CaCl_2_, 1 mM MgCl_2_, 1 mM NaH_2_PO_4_, pH 7.4, supplemented with 25 mM (PF25) or 280 mM glucose (PF280) as lyoprotector.

### 2.3. Biological Functionality In Vitro

N1E-115 murine neuroblastoma cell line was obtained from ATCC (CRL-2263, Manassas, VA, USA). This is a suitable target for NTS-polyplex NPs transfection because of their high density of NTSR1 [27,28]. These cells were cultured on 1 mm glass coverslips in DMEM supplemented with 10% FBS and incubated under 37 °C and 5% CO_2_. NTS-polyplex NPs in either EF (*n* = 6) or PF (*n* = 6) was added to the cell cultures when reached 50% of confluence. After 1 h incubation at 37 °C and 5% CO_2_, the transfection medium was withdrawn and substituted with DMEM containing 10% FBS. Cell cultures were incubated for additional 48 h. Upon completion of incubation, the cells were fixed with 4% paraformaldehyde and the nuclei were stained with 1 μM Hoescht-33258. The coverslips were mounted on glass slides using 1.5 μL VECTASHIELD (Vector Laboratories, Burlingame, CA, USA) and then sealed with nail polish. To qualitatively demonstrate GFP fluorescence, we used a Leica DMIRE2 microscope (Leica Microsystems, Wetzlar, Germany) with the filters A for Hoescht-33258 and K3 for GFP. The images were digitized with a Leica DC300F camera (Leica Microsystems, Nussloch, Germany). To quantify the transfection efficiency, we used a Flow cytometry at 519 nm emission (FL1) on a FACSScan Cytometer (Beckton Dickinson, Franklin Lakes, NJ, USA) equipped with an Argon Laser (488 nm). Previously, the cells were harvested with Pucks solution, then washed and re-suspended with PBS (*n* = 6 for each variable). Statistic *Student*’*s t* analyses were made using Statgraphics Centurion XVII software (Statgraphics Technologies, Inc., The Plains, VA, USA, 2017).

### 2.4. Biological Functionality In Vivo

#### Animals

Male Wistar rats (body weight of 210–230 g) were used and maintained under 12-h light-dark cycles at a controlled temperature (20–22 °C), 55% humidity and free access to food and water. The experimental protocol was approved by the Institutional Animal Care and Use Committee of CINVESTAV (authorization No. 162-15) certified by the Secretaría de Agricultura, Ganadería, Desarrollo Rural, Pesca y Alimentación (SAGARPA; NOM-062-ZOO-1999 and NOM-087-ECOL-1995). All efforts were made to minimize animals suffering and the number of animals used was kept to a minimum.

Rats were anesthetized with an intraperitoneal (i.p.) injection of anesthetic mixture (ketamine, 120 mg–xylazine, 9 mg, per kg of body weight) in 0.9% of saline solution (Pisa Agropecuaria, GDL, Mexico) and placed in a stereotaxic apparatus model 51600 (Stoelting, Wood Dale, IL, USA). Two microliters of either NTS-polyplex NPs (*n* = 3 rats) or DMEM (Sham transfection; *n* = 3 rats) were injected at the following coordinates, AP, +2.5 mm from interaural midpoint; ML, +2.0 mm from the midline; DV, −6.7 mm from dura mater. The injection flow was 1 μL/6 min using a microperfusion pump model KDS100L (KD Scientific, Holliston, MA, USA). After surgery, rats received an antibiotic and analgesic and were individually housed in the animal facilities until their use.

### 2.5. Transmission Electron Microscopy

NTS-polyplex NPs samples prepared either in EF25, PF25, or PF280 at the concentrations of 6 nM pDNA, 5 μM KP, 144 nM NTS-carrier were mixed with one volume of 1.5% uranyl acetate. A drop of this mixture was transferred to copper grids of 100 mesh previously covered with 2% formvar solution and coated with a thin carbon film. This preparation was kept in the copper grid for 1 min and then rinsed with distilled water twice. Samples were examined with a JEOL-JEM-1400 transmission electron microscope (JEOL USA, Inc., Peabody, MA, USA) at 80 kV accelerating voltage.

### 2.6. Dynamic Light Scattering

NTS-polyplex samples were prepared either in EF25 (*n* = 6) or PF25 (*n* = 6) at the optimum molar ratio and transferred into a Malvern DTS1060 capillary cuvette for dynamic light scattering analysis on a Malvern Zetasizer Nanoseries 3600 (Worcestershire, UK). Measurements were performed by triplicate considering the viscosity and dispersant of the water and the choice “protein” was selected in “Materials Manager.” Size distribution by intensity was fitted to the correlation function by the Zetasizer software (Version 7.03, Malvern, WR, UK, 2013) algorithms. Statistic *Student*’*s t* analyses were made using Statgraphics software.

### 2.7. Electrophoretic Mobility

Zeta potential of NTS-polyplex samples were prepared either in EF25 (*n* = 6) or in PF25 *(n* = 6) at the optimum pDNA:PK:NTS—carrier molar ratio (1:1:24), as determined by Electrophoretic Mobility and Laser Doppler Velocimetry using a Malvern Zetasizer Nanoseries 3600 (Worcestershire, UK). Samples were loaded into a Malvern DTS1060 capillary cuvette. Each sample was run by triplicate considering water viscosity. “Protein” was selected in Material manager and Smoluchowski function was set at a value of 1.5. Statistic *Student*’*s t* analyses were made using Statgraphics software.

### 2.8. Circular Dichroism (CD)

Samples of NTS-polyplex NPs were prepared in PF25 at optimum molar ratio and placed in an spectrosil grade quartz cuvette with an optical path of 0.1 cm. Samples were analyzed with a CD spectropolarimeter (Jasco J815 CD; Easton, Talbot County, MD, USA). CD spectra were collected from 203 to 300 nm with a data pitch of 2 nm; a scanning speed of 50 nm/min; a 5 nm band width and a total of 3 scans were collected and averaged for each sample.

### 2.9. Lyophilization Protocol 

Upon completion of assembling, 2 mL glass vials containing 300 μL of NTS-polyplex NP suspension were immediately frozen at −80 °C for 3 h at atmospheric pressure to immobilize the NPs. The frozen vials were then introduced into a Labconco Benchtop Freeze Dry System (Labconco, Kansas City, MO, USA) previously frozen at −80 °C and submitted to the following lyophilization cycle: −50 °C for 1 h, −35 °C for 14 h (0.08 mbar pressure); 0 °C for 1 h (0.08 mbar pressure), 20 °C for 5 h (0.04 mbar pressure). After lyophilization, vials were stoppered under vacuum and stored at room temperature until rehydrated. Moisture content was determined by Karl Fischer method and pH was determined with indicator strips from Merck Millipore (Burlington, MA, USA).

### 2.10. Size Exclusion Chromatography Using a Radioactive Tag

^99m^Tc-radiolabeled KP (NH_2_-RKKRRQRRRGGC[c(RGD-_D_Y-K)-3-succinimidopropionylamide]-G-C(Acm)-G-C(Acm)-CONH_2_(N_2_S_2_-RKKRRQRRR-c(RGDyK))), was obtained from Dr. Ferro-Flores (ININ, México) to form radioactive NTS-polyplex NPs in the PF25 at the optimum molar ratio that were analyzed with a Waters HPLC System (Waters, Milford, MA, USA). It’s worth mentioning that this ^99m^Tc-KP (called TAT-RGD) was proven to have comparable properties (final concentrations and functionality) to those of KP used in the rest of experiments, as shown by electrophoretic mobility and transfection assays (data no shown). The stationary phase was a Protein-Pak 300 SW column and the mobile phase was water at a flow of 1 mL/min. To monitor the stability of the complex and the assembly of radiolabeled KP, pDNA and NTS-carrier, a Waters Photodiode UV-Vis detector was used in series to a gamma radioactivity HPLC detector (Nikyang, Hong Kong, China).

## 3. Results

### 3.1. Formulation of NTS-Polyplex NPs

Considering the parameters listed in Table 1, we developed a PF that could provide stability and long shelf life to NTS-polyplex NPs for an eventual use in clinics.

NTS-polyplex NPs were used freshly prepared in EF25 (DMEM with 25 mM glucose). EF25 is composed by 7 inorganic salts, 14 amino acids, 8 vitamins and 1 sugar. For human use, the Food and Drug Administration (FDA) has not approved as inactive ingredients 1 of these inorganic salt (ferric chloride), 1 amino acid (cysteine) and 6 vitamins (choline chloride, folic acid, *myo*-inositol, pantothenic acid, pyridoxine and thiamine) [29]. So, our first aim was to develop PFs that can provide a long-term storage and were suitable for clinical use. Two PFs were developed by adding either 25 mM (PF25) or 280 mM (PF280) glucose to a standard formulation comprised of NaCl, KCl, CaCl_2_, MgCl_2_ and NaH_2_PO_4_, at isotonic concentration and pH 7.4. We chose glucose because the mammalian brain depends on this monosaccharide as main source of energy and its metabolism is fine regulated in the brain. These characteristics make glucose ideally suited for intracerebral administrations of NPs in comparison to reducing disaccharides as sucrose.

According to previous reports on nonviral systems [16,17,23,24,30,31], PF280 and EF280 have the suitable glucose-DNA ratio and concentration that could provide stabilization to NTS-polyplex NPs.

Table 2 shows that glucose concentration directly affects the osmolarity, as reported previously by Anchordoquy et al. [26] and the concentration of salts in the PFs is osmotically balanced. EF280 and PF280 resulted almost twice more hypertonic than EF25 and PF25, including the physiologic saline solution.

### 3.2. Biological Functionality In Vitro and In Vivo

**Figure 1:**
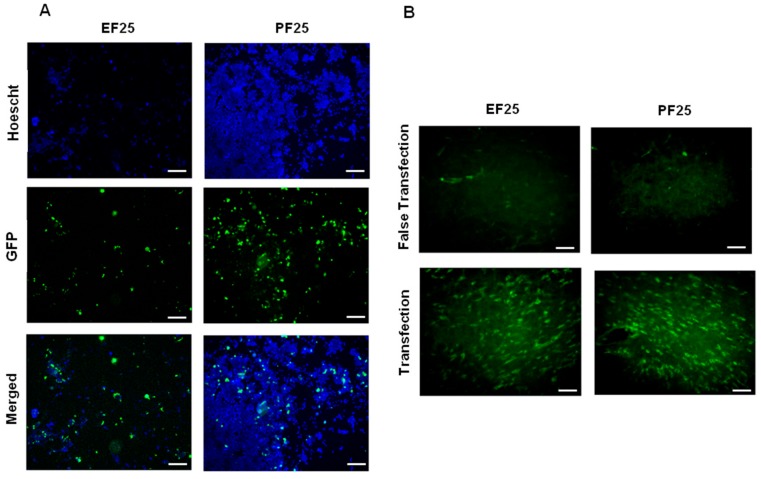
Effectiveness of NTS-polyplex NPs for EGFP-N1 transfection using EF25 and PF25 in vitro and in vivo. (**A**) Epifluorescence micrographs of Hoechst-counterstained N1E-115 cells expressing GFP; (**B**) Confocal micrographs of slices from s*ubstantia nigra* of Wistar rats with false transfection (injection of the respective vehicle) and with transfection. NTS-polyplex NPs were assembled with pEFGP-N1 in PF25 or EF25 at optimum molar ratio. Scale Bars = 150 μm.

To determine and compare the biological functionality in vitro of NTS-polyplex NPs assembled in EF and PFs, we carried out transfection assays with the plasmid pEFGP-N1 in N1E-115 cells and determined GFP expression by using epifluorescence microscopy and flow cytometry. In the case of EF280 and PF280, we observed cell death because of hypertonicity resulting from the high glucose concentration (Table 2), which was toxic and ruled out for future test. In contrast, NTS-polyplex NPs in EF25 or PF25 led to GFP expression in N1E-115 cells as shown by fluorescence microscopy (Figure 1A). The mean transfection efficiency was 22.13 ± 3.1% (EF25) and 27.92 ± 2.3% (PF25) as measured by flow cytometry (Supplementary Materials Figure S1). There was a significant difference determined by the parametric *Student*’*s t* test (*p* < 0.05; *n* = 6). These results demonstrate that in PF25, NTS-polyplex NPs exert a comparable and even higher transfection efficiency in vitro than in EF25. The increased transfection efficiency of NTS-polyplex NPs in PF25 can be caused by the low zeta potential (+14.1 mV) of this formulation as compared with that of EF25 (+25.3 mV). A low zeta potential reflects a higher electrostatic interaction between NTS-polyplex components (pDNA, KP and NTS-carrier). Thus, a higher interaction of these components might lead to a more efficient assembling of NTS-polyplex NPs and to a major availability for cell uptake. Thus, the saline composition and the glucose-DNA ratio of 241.8 in PF25 could confer adequate solubilization of NPs.

To further confirm the effectiveness of PF25 in vivo, we injected NTS-polyplex NPs containing the plasmid pEFGP-N1 into the *substantia nigra* of Wistar rats and compared the results with those from transfections using EF25. The confocal microscopy analysis at day 3 after transfection showed a large amount of *substantia nigra* cells that express GFP, in a similar fashion as with the transfection using EF25. In contrast, no GFP-expressing cells were observed in the *substantia nigra* injected only with the respective formulation (Figure 1B). These results are in agreement with those obtained in vitro and with previously published assays using EF25 [1,32]. Altogether, our results demonstrate that PF25 is an effective and safe vehicle for clinical use.

### 3.3. Lyophilization of NTS-Polyplex NPs

To confer more stability and long shelf life, a suitable lyophilization process for NTS-polyplex NPs in PF25 was developed, in which immediate immobilization of assembled NPs by freezing samples at −80 °C was the first step. The lyophilization cycle was developed considering the glass transition temperature for glucose (around −42 °C) [30] because this ingredient is the one that determines the lowest collapse temperature in PF25. For the concentration of glucose in PF25, the described conditions of freeze-drying (−50 °C for primary drying process; −35 °C for secondary drying process) gave an excellent cake. These same conditions applied to PF280 gave a viscous substance, suggesting a collapse due to the use of a higher temperature than *T_g_*.’ Regardless of this inconvenience, PF280 was not tested in biological assays after lyophilization because of its incompatibility with cell viability.

A cake of fine white powder was obtained in lyophilisates. Humidity determined by Karl Fisher method was <1%; reconstitution mean time was 9 s; and pH of reconstituted lyophilisates was 7.5 (*n* = 10). Then, we evaluated the lyoprotective ability of PF25 by determining biological functionality and physical properties of reconstituted NTS-polyplex NP lyophilisates. Lyophilisates were reconstituted with water for injection after 2 days of storage at 4 °C and 60% of relative humidity.

### 3.4. Effect of Lyophilization on Biological Activity of NTS-Polyplex NPs In Vitro and In Vivo

**Figure 2:**
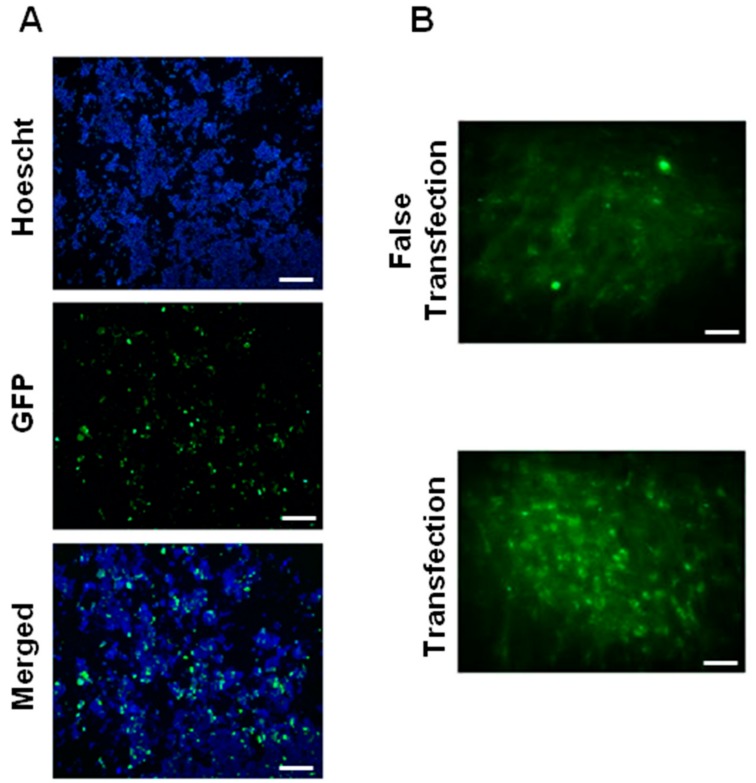
Biological functionality and transfection efficiency of reconstituted NTS-polyplex NPs in vitro and in vivo. (**A**) Epifluorescence micrographs of Hoechst-counterstained N1E-115 cells expressing GFP; (**B**) Confocal micrographs of slices from *substantia nigra* of Wistar rats. False transfection: injection of reconstituted PF25. NTS-polyplex was assembled with pEFGP-N1 in PF25 at optimum molar ratio, after lyophilization and reconstitution with water for injection after 2 days of storage at 4 °C. Scale Bars = 150 μm.

To evaluate the effectiveness of PF25 to maintain the functionality in vitro of reconstituted lyophilisates of NTS-polyplex NPs, transfection assays in N1E-115 cells were made. The results were compared with EF25 freshly prepared. Epifluorescence microscopy analysis showed a significant population of N1E-115 cells that express GFP when transfected with NTS-polyplex NPs from reconstituted lyophilisates or freshly prepared in EF25 (Figure 1A and Figure 2A). The transfection efficiency determined by flow cytometry analysis was 34.78 ± 3.96% for reconstituted NTS-polyplex NPs (Supplementary Materials Figure S2). There was significant difference (*p* < 0.05; *Student*’*s t* test; *n* = 6) when compared with the transfection efficiency of freshly prepared NTS-polyplex NPs. These results demonstrate that NTS-polyplex NPs in PF25 conserve their transfection ability after reconstitution of lyophilisates, even with higher transfection efficiency than freshly prepared NTS-polyplex NPs.

In agreement with the results in vitro, confocal microscopy analysis showed that a single injection of reconstituted NTS-polyplex NPs caused GFP expression in a great population of rat *substantia nigra* cells (Figure 2B) when compared with that caused by freshly prepared NTS-polyplex NPs in EF25 (Figure 1B). No GFP expression was seen in the *substantia nigra* with false transfection (administration of PF25 reconstituted with water). All these data show that 25 mM glucose in EF25 and PF25 is an efficient stabilizer and lyoprotectant for NTS-polyplex NPs.

### 3.5. Physical Structure of NTS-Polyplex NPs

**Figure 3:**
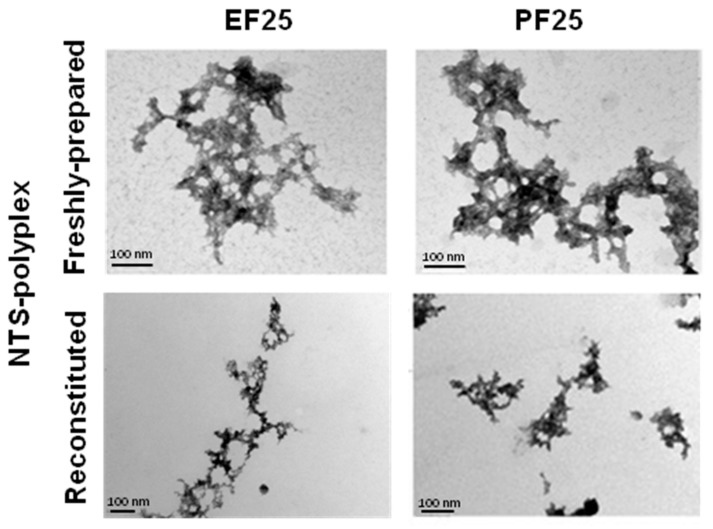
Electron Microscopy characterization of NTS-polyplex NPs prepared in different formulations and subjected to lyophilization. Lyophilisates of NTS-polyplex NPs were reconstituted with water after 2 days of storage at 4 °C.

To evaluate the structural integrity of NTS-polyplex NPs after lyophilization, TEM was used. Individual fresh samples of NTS-polyplex NPs prepared either in EF25 or in PF25 presented a typical toroid shape that results from the compaction degree of all components of NTS-polyplex NPs as reported previously for other polyplexes [19,33,34,35,36,37]. In addition, those NPs have an approximate diameter of 100 nm and a strong tendency to agglomerate forming complexes of up to a micrometric size. Reconstituted NTS-polyplex NPs display similar physical structure and size comparable to freshly prepared samples. However, the agglomeration pattern was altered, since NPs appear more disperse (Figure 3).

These results suggest that PF25 and the lyophilization process contributed to the protection, stability and dispersion of NTS-polyplex NPs.

### 3.6. Size and Z-Potential of NTS-Polyplex NPs

To confirm the size of NTS-polyplex NPs freshly prepared in EF25 and PF25 or reconstituted after lyophilization, Dynamic Light Scattering (DLS) was used. In addition, the zeta potential of the same samples was determined by measuring electrophoretic mobility using a Laser Doppler Velocimetry. The mean diameter of NTS-polyplex NPs freshly prepared in EF25 was 142.8 nm and the mean zeta potential was 25.3 mV (*n* = 6). NTS-polyplex NPs in PF25 had a mean diameter of 141.7 nm and a mean zeta potential of 14.1 mV (*n* = 6). There was no significant difference in the size of NPs in both formulations (*p* > 0.05; *Student*’*s t* test). However, there was significant difference in zeta potential of NTS-polyplex NPs in PF25 when compared with that in EF25 (*p* < 0.05; *Student*’*s t* test*; n* = 6) (Supplementary Materials Figure S3). This could result from the higher interaction of pDNA and NTS-carrier given by the ionic strength of PF25 in comparison to EF25, which also confers charges by its amino acids content. The mean diameter and zeta potential of reconstituted NTS-polyplex NPs in PF25 (*n* = 6) was 138.5 nm and 0.2 mV, respectively. There was significant difference in zeta potential of freshly prepared and reconstituted NPs (both in PF25) (*p* < 0.05; *Student*’*s t* test). In contrast, there was no significant difference in particle size (*p* > 0.05; *Student*’*s t* test) when compared with freshly prepared NTS-polyplex NPs (Supplementary Materials Figure S3). However, the mean polydispersity index (PdI) regarding size distribution of EF25 and PF25 freshly prepared samples was 0.59 and 0.55 respectively with no significant difference (*p* > 0.05; *Student*’*s t* test); whereas mean PdI for reconstituted NTS-polyplex NPs (PF25) was 0.47. There was significant difference with PdI of freshly prepared NPs in PF25 (*p* < 0.05; *Student*’*s t* test), which suggests a more homogeneous population and thus a higher stability of reconstituted NTS-polyplex NPs; this correlates with TEM results. On the other hand, a peak of 10 nm was observed in freshly prepared NPs, either in EF25 or PF25, which could be attributed to free NTS-polyplex components, as it was absent in the reconstituted samples (PF25). The intensity of reconstituted NPs distribution curve also increased five-fold (Supplementary Materials Figure S3). The near-to-zero decrease of zeta potential in these samples correlates with the absence of the putative free NTS-polyplex components. Probably they associate during reconstitution, leading to increased charge neutralization and thus, an increased number of suspended NPs.

### 3.7. Secondary Structure of NTS-Polyplex NPs

Secondary structure of freshly prepared and reconstituted NTS-polyplex NPs was determined using CD. This procedure could be useful for quality control due to its simplicity, quickness and low cost. PF25 facilitated the characterization of band patterns of NTS-polyplex NPs because this vehicle does not contain the components of EF25 that interfere with this spectroscopic analysis.

**Figure 4:**
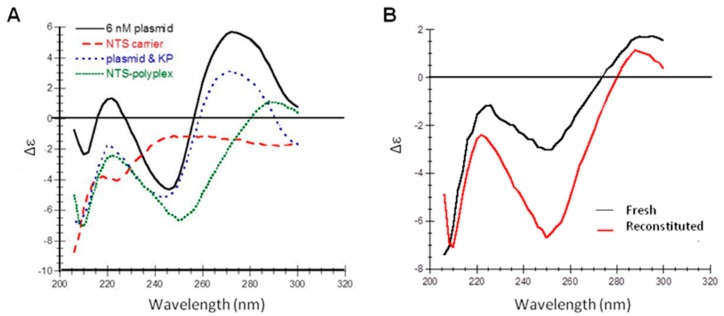
Circular Dichroism spectra of NTS-polyplex NPs and their components. (**A**) Merged spectra of pEGFP-N1 plasmid, NTS-carrier, pDNA-KP complex and NTS-polyplex NPS in PF25; (**B**) Spectra of newly assembled and reconstituted NTS-polyplex NPs in PF25.

In the first place, we confirmed that the band pattern in pDNA spectrum was similar to that previously reported [38,39]. The spectrum of the NTS-carrier resembles that of a random coil structure (Figure 4A).

The displacement of bands in the spectra of both pDNA-KP and NTS-polyplex NPs reveals the electrostatic interactions; firstly, between pDNA and KP and then, between pDNA-KP complex and NTS-carrier (Figure 4A). Interestingly, the spectra showed the similarity of reconstituted lyophilisates with fresh preparations and the conservation of the bands at 220, 250 and 290 nm after reconstitution (Figure 4B). The difference in intensity of elipticity might be due to the reconstitution procedure and it could correlate with particle size and zeta potential.

### 3.8. Stability of Reconstituted NTS-Polyplex NPs after Interaction with Serum

**Figure 5:**
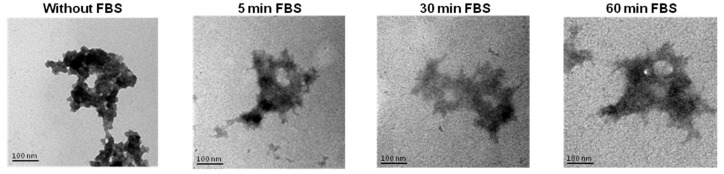
TEM images at different times of interaction of NTS-polyplex NPs with fetal bovine serum (FBS). Micrographs show NTS-polyplex NPs without and after 5, 30 and 60 min of interaction with FBS.

Serum proteins are the first biomolecules that interact with NTS-polyplex NPs when used in gene therapy protocols and therefore can affect the physical features in detriment of their transfection efficiency. Therefore, we used TEM to monitor the stability of reconstituted NTS-polyplex NPs in PF25 at different times of interaction (0, 5, 30 and 60 min) with FBS (Figure 5).

These micrographs show that reconstituted NTS-polyplex NPs conserve their physical integrity and no large aggregates were formed even after 60 min of interaction with FBS.

**Figure 6:**
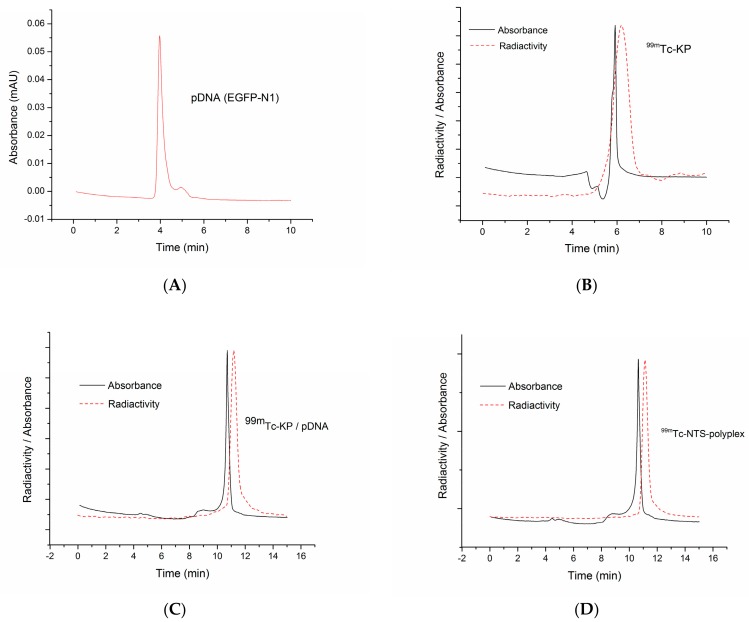

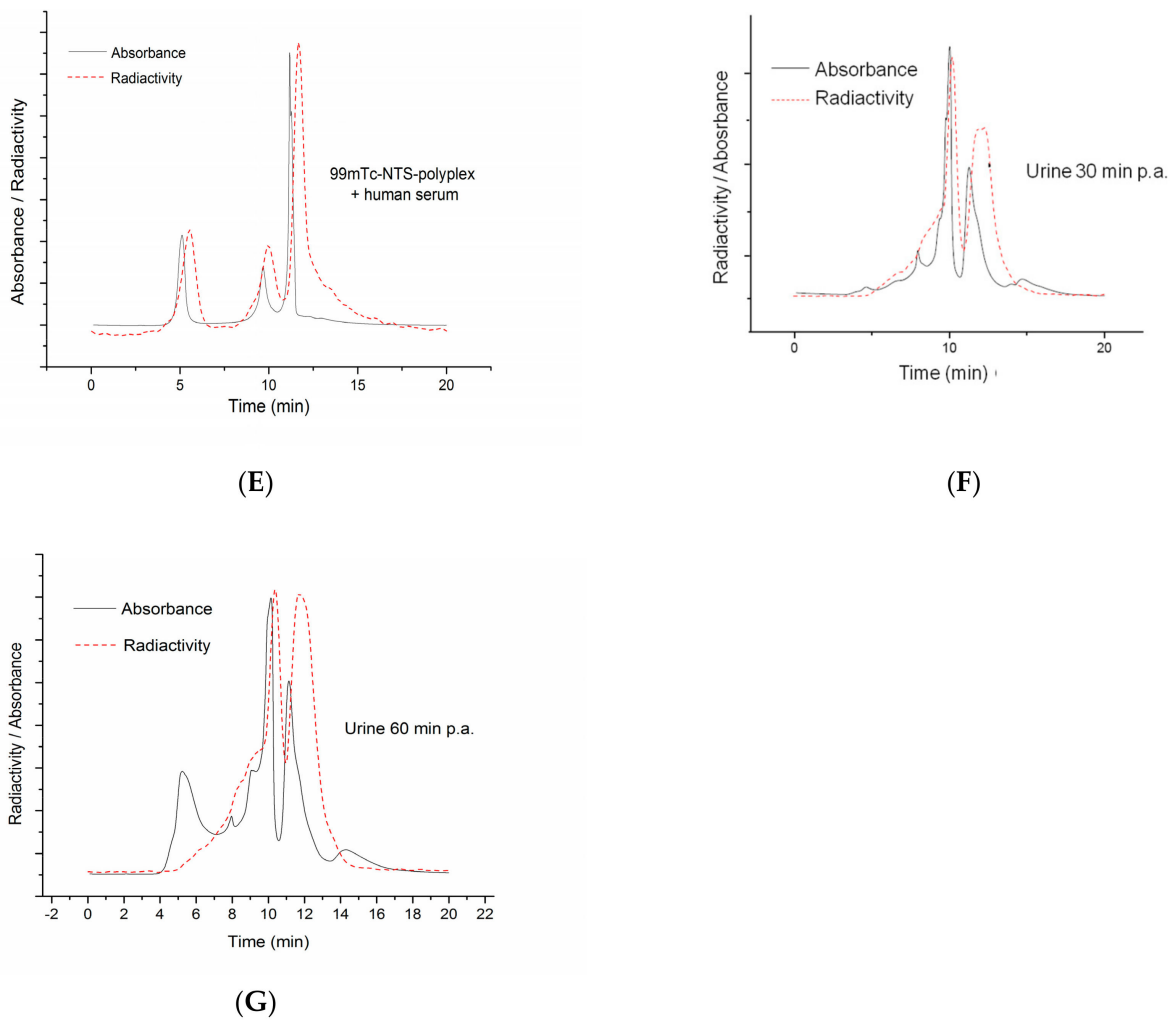
Chromatograms of SEC-HPLC for radiolabeled NTS-polyplex NPs and their components. (**A**) pDNA (pEGFP-N1); (**B**) ^99m^Tc-labeled Karyophilic Peptide (^99m^Tc-KP); (**C**) ^99m^Tc-KP-pDNA complex; (**D**) pDNA-^99m^Tc-KP-NTS carrier complex (^99m^Tc-NTS-polyplex NPs); (**E**) ^99m^Tc-NTS-polyplex NPs after 15 min interaction with human serum; (**F**) NTS-polyplex NPs in urine 30 min p.a.; (**G**) NTS-polyplex NPs in urine 60 min p.a. ^99m^Tc = ^99m^Technetium; p.a. = post-administration.

We used SEC-HPLC to further confirm the integrity of reconstituted radiolabeled NTS-polyplex NPs in PF25 after interaction with human serum. Firstly, we separately analyzed samples of pEGFP-N1, ^99m^Tc-KP, pEGFP-N1-^99m^Tc-KP complex and ^99m^Tc-NTS-polyplex in PF25 (Figure 6A–E). The retention time for pDNA (pEGFP-N1) was 3.97 min at 260 nm (Figure 6A) and for ^99m^Tc-KP was 5.91 min at 220 nm (Figure 6B). In the cases of pDNA-^99m^Tc-KP and ^99m^Tc-NTS-polyplex complexes, the retention times increased to 10.7 min (Figure 6C) and 10.9 min, respectively, at 295 nm (Figure 6D). These increases in retention times and the coincidence of the radioactivity peaks with the absorbance peaks are attributed to the electrostatic interactions of pDNA with ^99m^Tc-KP and then with the NTS-carrier. These interactions caused a super-compaction of pDNA into NPs that could longer interact with the column bed (Figure 6C,D).

The chromatogram at 295 nm for ^99m^Tc-NTS-polyplex after 15-min interaction with human serum showed three peaks (Figure 6E). The two small peaks were observed at 5.1 min and 9.63 min, whereas the high peak was at 10.8 min (Figure 6E). The latter peak corresponds to intact NTS-polyplex NPs as compared with the unexposed control (Figure 6D) and the other two populations of higher molecular mass might represent complexes of NPs with serum proteins. Moreover, we analyzed urine samples corresponding to 30 and 60 min post-administration to assess the presence of radioactive components or NTS-polyplex NPs. It was detected in both cases a peak of absorbance and radioactivity with a retention time of 10 min, suggesting the presence of components of NTS-polyplex NPs in urine (Figure 6F–G). In summary, the results from two independent techniques suggest that reconstituted NPs in PF25 might conserve their integrity in the bloodstream.

### 3.9. Stability Tests of Lyophilisates of NTS-Polyplex NPs in PF25

**Figure 7:**
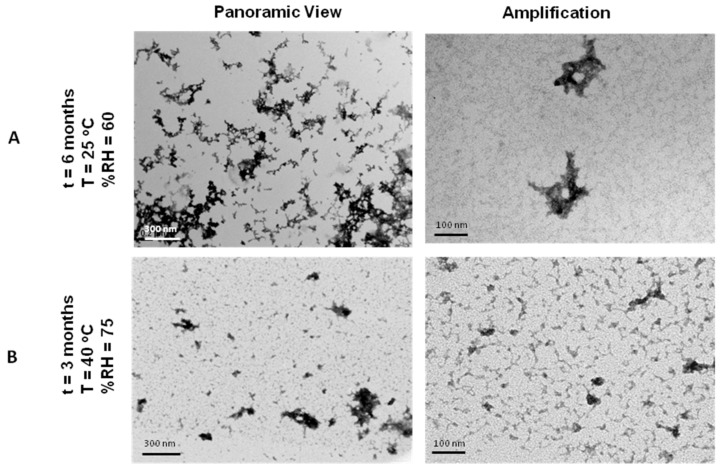
TEM micrographs of reconstituted NTS-polyplex lyophilisates in PF25 stored under different conditions of temperature and relative humidity (RH). (**A**) 6 months, 25 °C, 60% RH; (**B**) 3 months at 40 °C, 75% RH.

The accelerated stability test of NTS-polyplex NPs in PF25 was assessed through their shape and transfection efficiency. TEM analysis showed scarce toroid structures and abundant debris in reconstituted samples after 3 months of storage at 40 °C and 75% RH (Figure 7A) that failed to transfect N1E-115 cells, suggesting degradation.

On the contrary, lyophilisates of NTS-polyplex NPs in PF25 endured conditions of 25 °C and 60% RH for 6 months (Figure 7B), although their transfection efficiency decreased as compared with that of newly lyophilized NTS-polyplex (PF25) NPs (significant difference; *p* < 0.05, *Student*’*s t* test; (*n* = 6)) (Supplementary Materials Figure S4). NTS-polyplex NPs kept their shape and biological functionality after reconstitution and storage a 25 °C and 60% RH.

Thus, these results demonstrate that PF25 is a suitable vehicle of NTS-polyplex NPs for gene therapy applications in the clinic.

## 4. Discussion

Despite the advances in academic research, the clinical translation of nanovectors remains challenging, particularly from the manufacturing and regulatory perspectives. The transition of nanovectors from encouraging preclinical research to clinical trials and finally regulatory approval has not yet occurred.

The dimensions and structural complexity of NTS-polyplex NPs require specific characterization and analytic techniques, as well as a robust manufacturing process to ensure the production of high quality, safe and effective products.

The preclinical success in the treatment of Parkinson’s disease and cancer [6,9,10] and the results herein described support the use of NTS-polyplex NPs in the clinic. Our results demonstrate that the maintenance of particle size was achieved after lyophilization, indicating that the typical tendency of nanovectors to form aggregates over time was overcome. The conservation of shape and transfection efficiency of lyophilisates even after long-term storage under the appropriate conditions is also closely related to particle size maintenance, since intermediate (100–200 nm) ligand-coupled polylysine and PEI complexes primarily enter through clathrin-coated pits. In contrast, larger complexes (>200 nm) can be unspecifically internalized by macropinocytosis [40] or cleared by the reticulate endothelial system [12]. Aggregation finally leads to a decreased transfection efficiency [16,18,23,31,41]. 

Besides lyophilization, it is important to consider the effect of ingredients of a formulation on the nanovectors properties. For example, low salt concentration in PF25 contributes to decrease aggregation of NTS-polyplex NPs. It has been reported that increased salt concentration reduces the hydrate layer around the particles and promotes their aggregation [18]; and a high ionic strength produces significantly larger toroids [42].

The use of lyoprotectants like saccharides in the formulation is essential to conserve NPs structure and functionality [18,23,43]. The advantage of using glucose over other saccharides is that it is a component of cerebrospinal fluid and plasma, thus making the vehicle more tolerable when used intracerebrally for Parkinson’s disease treatment or intravenously for cancer treatment. In accordance to the water replacement theory, at the concentration used in PF25, glucose forms hydrogen bonds to the surface of NTS-polyplex NPs and helps to retain native structure in the dried state (maybe even after reconstitution, avoiding agglomeration). The glassy matrix theory, in which lyoprotection is mainly given by a viscous matrix formed by highly concentrated saccharides, is here ruled out [24,25,26,43]. In the case of NTS-polyplex NPs, a low glucose/DNA ratio, in comparison with other polyplexes and saccharides [17,23,44], was enough to confer cryo- and lyoprotection and to maintain the isotonicity of PF25. This low and enough glucose/DNA ratio is given and/or explained by: (1) the saccharide used; (2) the characteristics of NTS-carrier; (3) the low NPs dose that has been demonstrated to be therapeutically efficient [4,9,10]. It has been considered a dose adjustment for human use in a volume but not in the concentration, so this third point has been overcome.

It has also been reported that polyplexes with neutral surface charge are more instable, aggregate more easily in solution and form precipitates [45,46,47]. NTS-polyplex NPs in PF25 showed a significant decrease in zeta potential as compared with NPs in EF25. To avoid modifying N/P ratio in NTS-polyplex NPs (because of undesirable effects of a strong positive or negative surface charge in vivo; see below), we overcame this drawback with the immobilization of NPs during the freezing step of lyophilization, as aggregation was not observed during incubation at temperatures below *T*’*g* [48]. This was achieved by immediately freezing NPs after the assembly in PF25 and incubating the vials at −80 °C (temperature below *T*’*g*) for 3 h prior to the sublimation phase of lyophilization. This also counterbalanced the absence of glassy matrix formed by high (hypertonic) concentration of saccharides, which also has the role of immobilizing NPs during the freezing step of lyophilization.

Thus, the designed lyophilization protocol, in concert with glucose included in PF25, was able to preserve the physical characteristics of NTS-polyplex NPs, as demonstrated by secondary structure and particle size analyses. The freshly prepared NTS-polyplex NPs (both EF25 and PF25-formulated) showed a PdI quite large (0.59 and 0.57, respectively), which resulted from a bimodal particle size distribution (Supplementary Materials Figure S3). One peak represents a population with a mean diameter of about 10 nm, which is attributed to free NTS-polyplex components. Another peak represents a larger population with mean diameters of about 142 nm, which corresponds to NTS-polyplex NPs. The low zeta potential values in PF25 (+14.1 mV), which reflects an efficient pDNA compaction because of the neutralization of NTS-polyplex components, may account for the decrease in the intensity of 10 nm population (free components) and the increase in the intensity of 142 nm population (NPs) with respect to the corresponding populations in EF25. Particle size distribution could be well controlled by the lyophilization process as shown by the decreased PdI (0.47) and the unimodal distribution of the reconstituted NTS-polyplex NPs (Supplementary Materials Figure S3). These results correlate with results from TEM and zeta potential analysis. NPs presented a lower tendency to aggregate after reconstitution and showed a zeta potential mean value of 0.2 mV, which reflects the high efficiency of NPs assembly and consequently the disappearance of the 10 nm-peak population. The critical parameters that may affect the particle size and size distribution of the resulting NPs are the following: (1) salt concentration in formulation [19,42]; (2) mixing conditions of the components; (3) immediate immobilization of NPs once assembled and (4) lyophilization process and reconstitution.

Lyophilization also contributed to the increase in transfection efficiency of NTS-polyplex NPs in PF25 as compared with that in freshly prepared NPs. Stability, dispersion and neutralization of those NPs are features of reconstituted lyophilisates as shown by TEM micrographs and zeta potential results. These features can be attributed, in the first place, to the immediate immobilization of NTS-polyplex NPs by the freezing after assembling. In addition, the replacement of water molecules by glucose functional groups in lyophilization might conserve the structure and prevent agglomeration of NTS-polyplex NPs. Finally, the efficient electrostatic neutralization of NTS-polyplex components might also avoid their agglomeration after reconstitution.

Once administered intravenously, polyplex aggregation and disassembly might be caused by unwanted interactions with blood serum, biological tissue and immune cells. Increase in size of polyplex systems due to aggregation in systemic circulation could lead to capillary bed capture in the location or first encounter and restrict polyplex migration across size-dependent endothelial gaps [43]. Surface charge plays a major role in size maintenance and functionality of NPs in vivo. A strong positive charge on the NPs facilitates unspecific interactions with negatively charged proteins of plasma, extracellular matrix and cell surface, whereas strong negative charges can be scavenged by phagocytosis via the macrophage polyanion receptor [49]. Thus, stabilization of superficial charge of polyplexes should confer enough time of bloodstream circulation to allow the targeting to specific cell types by utilizing the interactions between surface receptors and ligands. Reconstituted NTS-polyplex NPs have practically neutral surface charge, so it could explain the results of NPs after their interaction with FBS or human serum. Physical integrity was conserved even after 60 min of interaction with FBS as revealed by TEM. Similar results were obtained after incubation of ^99m^Tc-NTS-polyplex NPs with human serum, as shown by SEC-HPLC analysis. However, after 30 min of interaction, two small fractions of higher molecular weight were separated. Three hypotheses can explain the presence of these two populations. The first hypothesis is that the ionic strength of human serum promotes the self-aggregation of NTS-polyplex NPs. The second hypothesis is that these NPs interact with human serum proteins to form high molecular weight aggregates. And the third hypothesis is that NTS-polyplex NPs undergo conformational change or degradation after interaction with human serum proteins or due to its ionic strength. Spectroscopic and chromatographic analysis beyond the scope of this work can provide further insight into the nature of these populations in order to propose a model for the dynamics of NTS-polyplex NPs after systemic administration.

Urine clearance of components of ^99m^Tc-NTS-polyplex was demonstrated by SEC-HPLC at 30 and 60 min after administration, as shown by the peak corresponding to the retention time of 10 min. Considering that only the disassembly of NPs at the kidney glomerular basement membrane would allow transit of the NPs components into the urine, a hypothesis that could explain our findings is that NPs reform in the urine after disassembly and filtration, as demonstrated by Zuckerman et al. [50], suggesting that at least a population of excess NPs arrives to the kidneys in an assembled and non-aggregated way. However, the decrease in the retention time might be due to a reduction in the interaction of components that provide a higher level of compaction to pDNA during re-assembly conditions. The peak at 12.5 min at 295 nm that presents a high level of radioactivity could be explained by the degradation of free radioactive KP in urine. Anyhow, further biodistribution studies of PF25-formulated NTS-polyplex should be carried out.

Finally, accelerated stability tests of NTS-polyplex lyophilisates in PF25 demonstrated that these preparations remain stable and functional, with transfection efficiency comparable to freshly prepared NTS-polyplex NPs, for at least 6 months at 25 °C and 60% relative humidity. Degradation of NTS-polyplex NPs after 3 months at 40 °C and 75% RH can be attributed to molecular mobility in these conditions. Previous reports have shown that the storage stability of amorphous pharmaceuticals in the solid state is largely affected by changes in molecular mobility at *T_g_*, making the storage temperature a determinant factor for the chemical and physical degradation rates. Physical degradation rates of pharmaceuticals, such as crystallization of amorphous compounds, are also related to molecular mobility. In addition, aggregation, one of the most common degradation cause of lyophilized biomolecule formulations, involves collisions between molecules and is closely related to molecular mobility [51]. Although our lyophilisates showed less than 1% humidity, the storage at temperatures above *T_g_* may have led to chemical and physical degradation. The decrease in transfection efficiency after 6 months of storage at 25 °C and 60% RH can be attributed to a probable molecular mobility after a long storage time, even though storage temperature and RH were adequate. An accelerated stability study suggests that in tropical weathers, it would be recommended to storage lyophilisates under refrigeration. All these findings suggest that PF25 is an appropriate vehicle for the clinical use of NTS-polyplex NPs that also confers a considerable shelf life at room temperature after lyophilization.

## 5. Conclusions

This work describes analytical methods, instrumental techniques and a lyophilization process to develop a pharmaceutical formulation for NTS-polyplex NPs that showed to be safe and provide high transfection efficiency within a long shelf life. According to our findings, a glucose-pDNA ratio (*w*/*w*) as low as 250 was able to confer lyoprotection and stability to NTS-polyplex NPs, in concert with adequate conditions for freezing and/or lyophilization. Our experimental design could be applied to the clinical formulation of other polyplexes.

## Figures and Tables

**Table 1:** Components in formulation for polyplexes.

Component	Function	Examples	Selected Ingredient(s)	Reference
Stabilizer	Control of NPs size	**Salts**	NaCl, KCl, CaCl_2_, MgCl_2_, NaH_2_PO_4_	[19]
Buffer	Preserve pH balance	**Phosphates**, citrates, amino acids	NaH_2_PO_4_	[21]
Tonicity modifiers	Provide Isotonicity	**Salts, saccharides**, polyols, polymers	NaCl, glucose	[26]
Cryo-and lyoprotectant/Bulking agent	Avoid fracturing	**Saccharides**, polyols, polymers, amino acids	Glucose	[17,18,22,23,24,25,26]

**Bold:** selected groups or molecules for the prototype formulation (PF).

**Table 2:** Glucose-DNA ratio and osmolarity of different formulations for NTS-polyplex NPs.

Vehicle	Glucose-DNA Ratio	Osmolarity (mOsm/L)
PSS	Not applicable	289.2
EF25	241.8	283
PF25		280.6
EF280	2710.6	480.6
PF280		501.2

PSS, Physiological Saline Solution; EF, Experimental Formulation; PF, Prototype Formulation.

## Supplementary Materials

The following are available online at www.mdpi.com/1999-4923/10/1/5/s1. Figure S1: Representative graphics of flow cytometry analysis of N1E-115 cells transfected with NTS-polyplex NPs in EF25 or in PF25; Figure S2: Representative graphics of flow cytometry analysis of N1E-115 cells transfected with NTS-polyplex NPs formulated in PF25, lyophilized and reconstituted with water for injection; Figure S3: Representative graphics of size distribution and zeta potential of freshly prepared NTS-polyplex NPs in PF25 or in EF25 and NPs formulated in PF25, lyophilized and reconstituted with water for injection; Figure S4: Representative graphics of flow cytometry analysis of N1E-115 cells transfected with NTS-polyplex NPs formulated in PF25, lyophilized and reconstituted with water for injection after their storage for 6 months at 25 °C and 60% Relative Humidity.

Supplementary File 1
