# Supplementary material for: Development of a Parenteral Formulation of NTS-Polyplex Nanoparticles for Clinical Purpose
Source: Pharmaceutics. 2018 Jan 3;10(1):5. doi: 10.3390/pharmaceutics10010005 (PMC5874818; doi:10.3390/pharmaceutics10010005)
Supplement: Supplementary File 1 [file pharmaceutics-10-00005-s001.pdf]

Development of a Parenteral Formulation of NTS-Polyplex Nanoparticles for Clinical Purpose

María E. Aranda-Barradas [1], Maripaz Márquez [2,3], Liliana Quintanar [2], Jaime Santoyo-Salazar [4], Armando J. Espadas-Álvarez [5], Daniel Martínez-Fong [1,5,†] and Elizabeth García-García [6,*,†]

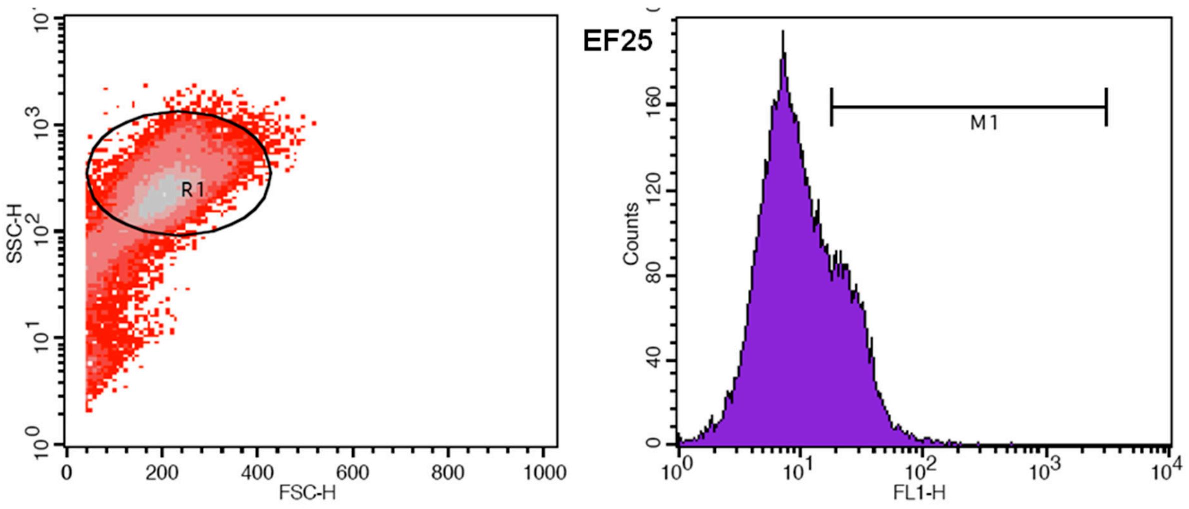

Marker	Left, Right	Events	% Gated	% Total	Mean	Geo Mean
All	1, 9910	27451	100.00	81.55	12.67	9.71
M1	18, 3078	5989	21.82	17.79	28.91	27.03

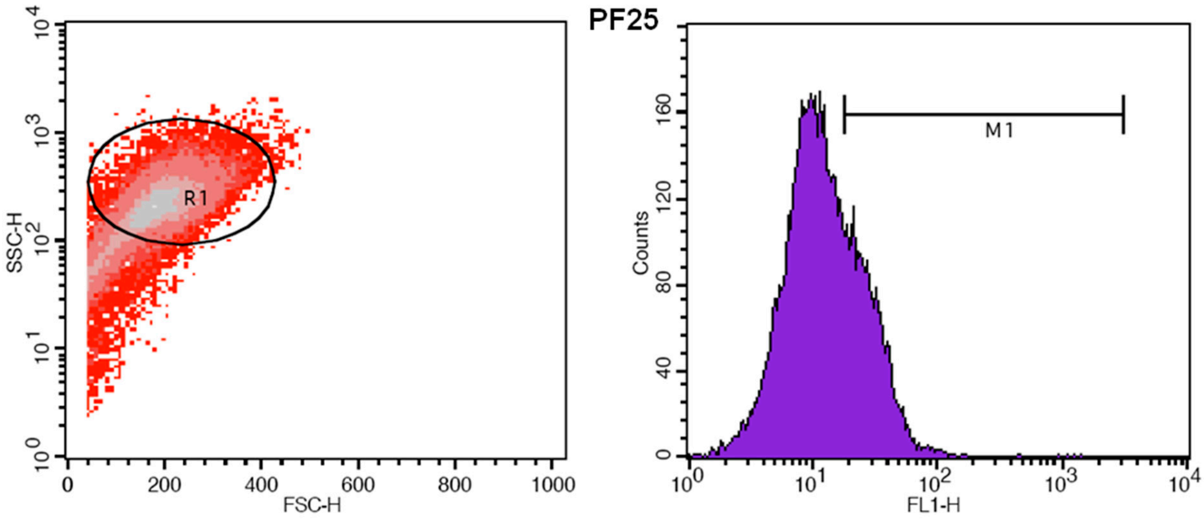

Marker	Left, Right	Events	% Gated	% Total	Mean	Geo Mean
All	1, 9910	26120	100.00	78.40	15.14	11.94
M1	18, 3078	7346	28.12	22.05	29.76	27.42

Figure S1. Representative graphics of flow cytometry analysis of N1E-115 cells transfected with NTS-polyplex NPs in EF25 or in PF25.

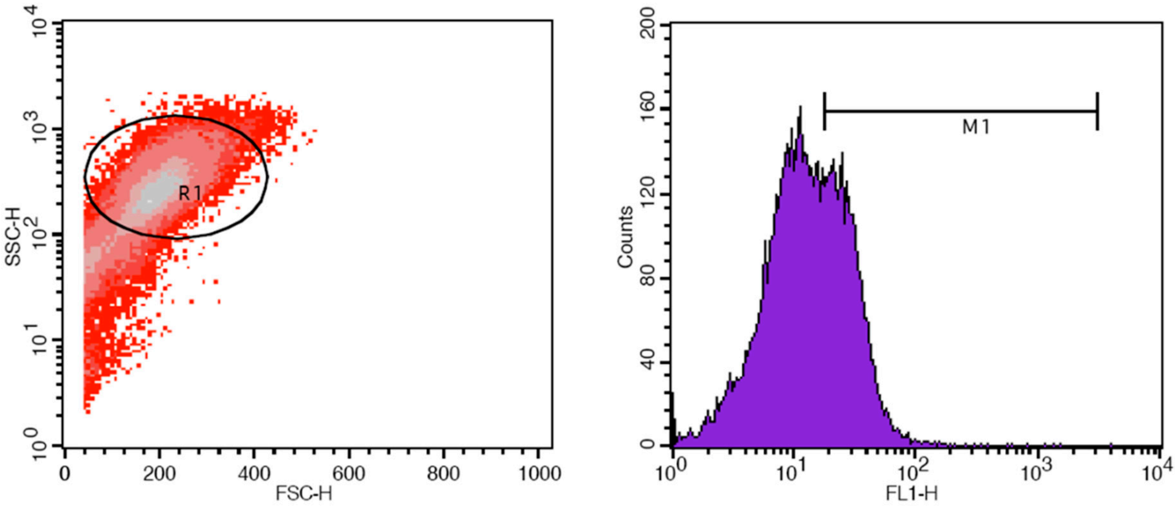

Marker	Left, Right	Events	% Gated	% Total	Mean	Geo Mean
All	1, 9910	27939	100.00	76.42	16.54	12.58
M1	18, 3078	9780	35.00	26.75	29.58	27.35

Figure S2. Representative graphics of flow cytometry analysis of N1E-115 cells transfected with NTS-polyplex NPs formulated in PF25, lyophilized and reconstituted with water for injection.

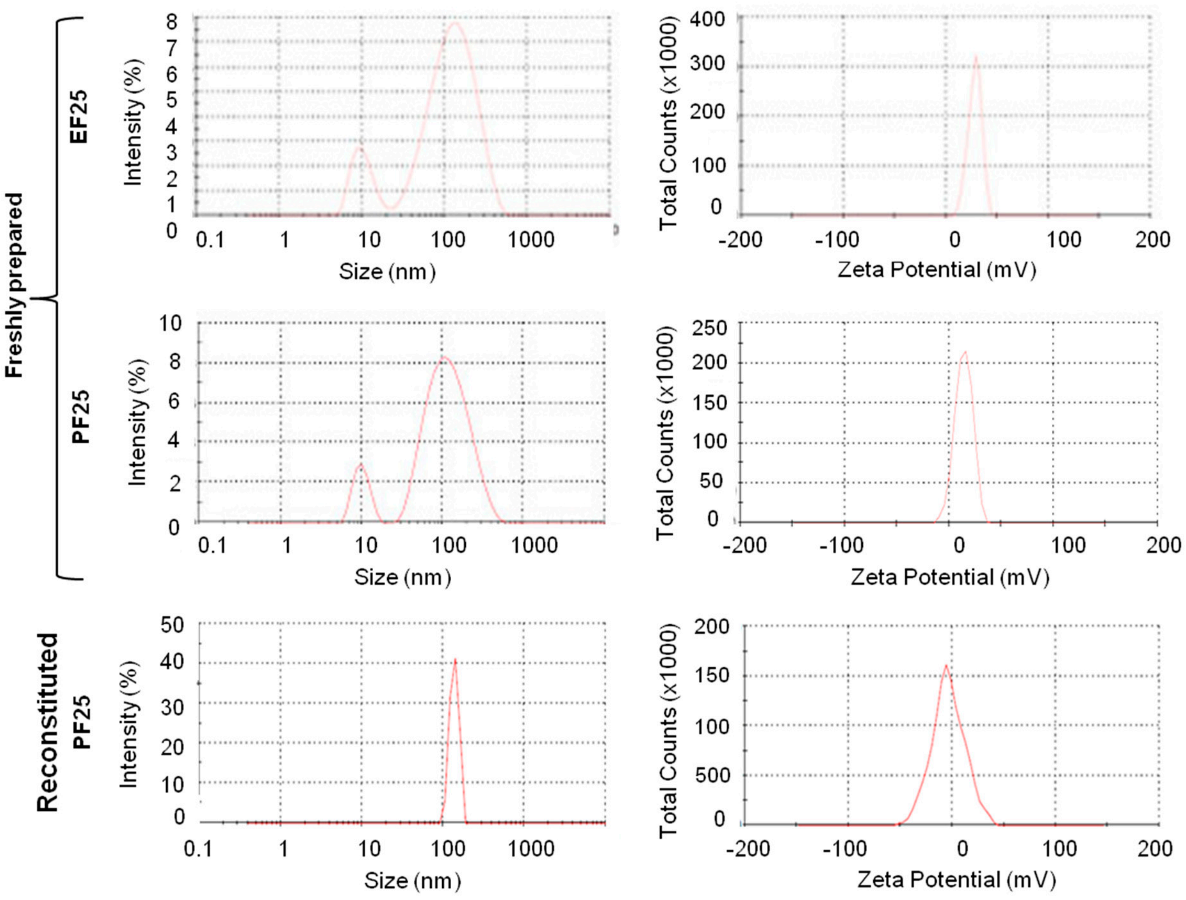

Figure S3. Representative graphics of size distribution and zeta potential of freshly prepared NTS-polyplex NPs in PF25 or in EF25 and NPs formulated in PF25, lyophilized and reconstituted with water for injection.

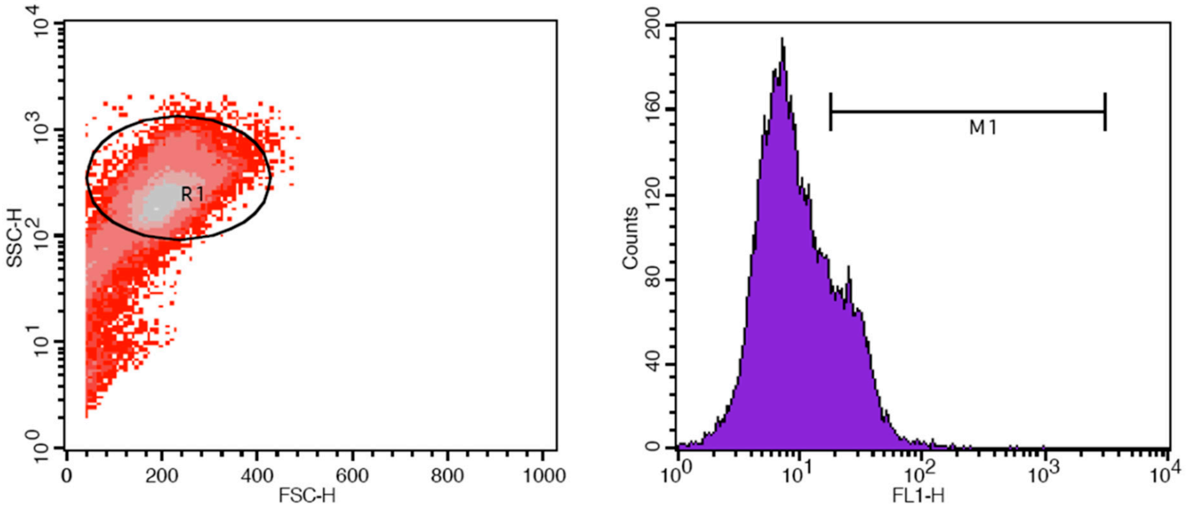

Marker	Left, Right	Events	% Gated	% Total	Mean	Geo Mean
All	1, 9910	27360	100.00	81.87	12.37	9.44
M1	18, 3078	5686	20.78	17.01	29.29	27.28

Figure S4. Representative graphics of flow cytometry analysis of N1E-115 cells transfected with NTS-polyplex NPs formulated in PF25, lyophilized and reconstituted with water for injection after their storage for 6 months at 25 °C and 60% Relative Humidity.
